# The Removal of Meat Exudate and *Escherichia coli* from Stainless Steel and Titanium Surfaces with Irregular and Regular Linear Topographies

**DOI:** 10.3390/ijerph18063198

**Published:** 2021-03-19

**Authors:** Adele Evans, Anthony J. Slate, I. Devine Akhidime, Joanna Verran, Peter J. Kelly, Kathryn A. Whitehead

**Affiliations:** 1 Faculty of Science and Engineering, Manchester Metropolitan University, Manchester M1 5GD, UK; Adele.Evans@mmu.ac.uk (A.E.); D.Akhidime@mmu.ac.uk (I.D.A.); j.verran@mmu.ac.uk (J.V.); peter.kelly@mmu.ac.uk (P.J.K.); 2 Department of Biology and Biochemistry, University of Bath, Claverton Down, Bath BA2 7AY, UK; ajs319@bath.ac.uk; 3 Microbiology at Interfaces, Department of Life Sciences, Manchester Metropolitan University, Manchester M1 5GD, UK

**Keywords:** *Escherichia coli*, bacterial retention, surface topographies, meat exudate, wipe cleaning, conditioning film

## Abstract

Bacterial retention and organic fouling on meat preparation surfaces can be influenced by several factors. Surfaces with linear topographies and defined chemistries were used to determine how the orientation of the surface features affected cleaning efficacy. Fine polished (irregular linear) stainless steel (FPSS), titanium coated fine polished (irregular linear) stainless steel (TiFP), and topographically regular, linear titanium coated surfaces (RG) were fouled with *Escherichia coli* mixed with a meat exudate (which was utilised as a conditioning film). Surfaces were cleaned along or perpendicular to the linear features for one, five, or ten wipes. The bacteria were most easily removed from the titanium coated and regular featured surfaces. The direction of cleaning (along or perpendicular to the surface features) did not influence the amount of bacteria retained, but meat extract was more easily removed from the surfaces when cleaned in the direction along the linear surface features. Following ten cleans, there was no significant difference in the amount of cells or meat exudate retained on the surfaces cleaned in either direction. This study demonstrated that for the *E. coli* cells, the TiFP and RG surfaces were easiest to clean. However, the direction of the clean was important for the removal of the meat exudate from the surfaces.

## 1. Introduction

Modern food processing and production facilities provide an environment that promotes bacterial retention due to a myriad of factors, which include the surface properties of the equipment and the matrix of the food being processed [1,2]. The removal of bacteria and/or organic material from food production surfaces is important since its build up can result in microbial contamination of food products, which can have a significant effect on consumers, food companies, and food suppliers, for example, cross-contamination of food with pathogenic bacteria can result in food-borne illnesses [3,4,5,6]. The Food Standard Agency estimates that foodborne illness in the UK alone result in a financial loss of £1.5 billion per annum [7]. As such, biofouling in the food industry is a significant problem [8]. For certain bacteria, some of which are important human pathogens, there can be contamination of raw meat due to biofouling [9]. Contamination of beef with *Escherichia coli* O157:H7 has been linked to outbreaks of foodborne illnesses and concerns about *E. coli* O157:H7 contamination have resulted in a zero tolerance towards this microorganism in the food industry [10,11].

Once a surface is introduced into an environment, it will adsorb a variety of organic and inorganic matter, resulting in the formation of a conditioning film [12,13,14]. Surface conditioning is a process which starts within seconds of the substratum becoming immersed into liquids [15]. The structure and composition of a conditioning film is ultimately dependent upon the surrounding products and the properties of the surface and can result in physicochemical, chemical, and topographical alterations, affecting both the rate and extent of bacterial retention and therefore surface contamination [16,17,18]. With regards to the meat industry, the exudate of frozen raw meat has been identified as an important source of bacterial contamination on food processing surfaces [19]. It has also been shown that sterilized chicken juice is an ideal environment for survival of *Campylobacter jejuni* [20] and its presence may also increase biofilm formation [21].

The method and type of physical cleaning methods used will be dependent on the food industry and the surfaces involved [22]. Product contact surfaces may typically be cleaned several times per day, while environmental surfaces such as walls and hoods may be cleaned less frequently [23]. In the meat industry, chilled beef carcasses are cut into smaller pieces, which are deboned and made into cuts; such work takes place on flat surfaces that are regularly cleaned [24]. However, it has been suggested that bacterial recontamination during this meat fabrication process results in higher numbers of *E. coli* on the cuts and trimmings [25,26]. Hence, a better understanding is required of the mechanisms involved in the attachment and detachment of bacteria to meat processing surfaces and their removal following cleaning. To simulate more realistic conditions, cleaning assays need to be carried out in the presence of a meat exudate (or relevant conditioning film) to increase the understanding of surface hygiene and decrease transmission and hence potential public health risks [27]. 

The ideal conditions for a hygienic surface have been defined as easy to clean, able to resist wear and maintain their hygienic qualities over time [28]. The hygienic quality and cleanability of a surface has been linked to the surface properties including the topography [28,29,30], chemical composition [31] and physicochemical properties [32,33]. Thermodynamics are thought to play a central role in initial bacterial: substrata interactions where it has been suggested that bacterial cells will attach preferentially to hydrophobic materials (i.e., materials with a low surface energy), when the surface energy of the bacteria is greater than the surface energy of the surrounding liquid [34]. Due to the complexity of bacterial-substratum interactions, further research is required to fully elucidate the underpinning mechanisms of bacterial attachment, adhesion, and retention [35]. 

An approach to reduce microbial contamination, which is a prerequisite for biofilm formation, is the modification of surface topography. Microscale surface topographic features have been shown to both inhibit or promote bacterial retention depending on the size, shape, and density of the topographical features [36]. It has also been shown that surfaces with features on the same scale as bacterial cells (e.g., cocci-shaped *Staphylococcus aureus*; ~1 µm diameter) promote the strongest retention due to maximum binding at the cell-substrate contact areas [37,38]. In an industrial setting, the wear of the surfaces may introduce random features (i.e., scratches) of different dimensions and it has been suggested that an increase in the surface roughness may cause the entrapment of microorganisms within the surface features, which in turn will affect the cleanability and hence the hygienic status of the surface [39]. Bacteria and organic material that become entrapped in the topographical features of a surface are difficult to remove using standard cleaning procedures [40], and it has been proposed that the development of the micro-pattern materials may help in the reduction of viable bacteria on food contact surfaces [41]. However, most studies have not determined the effect of the presence of the conditioning film on surface cleaning, especially with regards to the influence of surface topographical features [20], or with regards to the direction of cleaning compared to the linear surface features.

Stainless steels are used widely throughout the food and beverage industry due to their resistance to corrosion, thermal conductivity, and their ability to be produced with a smooth surface finish [33]. Stainless steel grade 304 is most commonly used in the food industry [42]. Due to the production process of stainless steel, ‘microniches’ of heterogenous chemical composition may result in varying bacterial retention patterns [43]. Titanium has been incorporated into stainless steel alloys in the food industry to improve corrosion resistance because it forms stable carbides [44,45]. Titanium surfaces may also have a more homogenous chemical composition than stainless steel since it is comprised mainly of TiO_2_ [46]. This work aimed to determine how surface attributes (chemistry and topography) and the direction of cleaning affected bacteria and meat exudate removal from surfaces. 

## 2. Materials and Methods 

### 2.1. Equipment and Material Suppliers

The following reagents and materials were used; stainless steel sheets (Outokumpu Stainless Ltd., Helsinki, Finland), sodium hydroxide, di-potassium hydrogen phosphate, potassium di-hydrogen phosphate, tri-sodium citrate ammonium sulphate, magnesium sulphate (Merck, Darmstradt, Germany), tryptone soya agar and tryptone soya broth (Oxoid, Basingstoke, UK) rolled beef brisket (Co-op, Manchester, UK), *Escherichia coli* CCL410 (Agence Francaise de Securite Sanitaire des Aliments, Paris, France), cleaning clothes (WYPALL^®^ ×80 Kimberley-Clark, West Malling, UK), Rhodamine B, DAPI and glycerol (Merck, Darmstradt, Germany). The following equipment was purchased: Atomic force microscope (Quesant Instruments, Santa Cruz, CA, USA), Crockmeter (A.A.T.C.C Crockmeter, Model CM1, NC, USA), Epifluorescence microscope (Nikon, Tokyo, Japan), F-View II camera (Soft Imaging System Ltd., Olympus, Tokyo, Japan), and Cell F Image Analysis package (Olympus, Tokyo, Japan).

### 2.2. Production of Surfaces

Three different surfaces were used in this study, including stainless steel 304 with a fine polished finish (FPSS), 304 fine polished finished stainless steel coated with titanium (TiFP) and a linear, regular finished (RG) titanium surface. Fine polished, grade 304, stainless steel sheets were prepared as 10 mm × 10 mm sample squares using a guillotine. To ensure that the samples were examined in a pristine “as-manufactured” state, the manufacturer’s protective plastic coating was only removed directly before experimentation. 

The titanium surfaces with a regular topography were unwritten digital video discs stripped of their protective coats. The samples were cut into 10 mm × 10 mm squares using metal cutting shears and soaked overnight in 30% sodium hydroxide solution, followed by rinsing thoroughly with sterile distilled water and drying in a class 2 microbiological cabinet prior to coating with titanium.

Samples of the fine polished stainless steel surfaces and the stripped digital video discs were coated using titanium. The substrata were coated with titanium via magnetron sputtering in a modified Edwards E306A coating system rig using a single 150 mm diameter × 10 mm thick, 99.5% pure titanium target attached to an unbalanced magnetron (argon gas at a working pressure of 0.15 Pa; magnetron power of 0.5 kW; base pressure 10^−4^ Pa; time 15 min; substrate biased at −50 V) [47].

### 2.3. Atomic Force Microscopy (AFM)

The shape and depth of the surface features was determined using atomic force microscopy. The analysis was carried out in in contact mode using triangular shaped silicon nitride tips, with a spring constant of 0.12 N m^−2^. The height and shape of the features were determined from five areas taken from different replicate surfaces.

### 2.4. Sample Organisms

This study was conducted with *Escherichia coli* strain CCL410. This strain was recovered by the laboratory of Dr C. Vernozy-Rozand (Unité de Microbiologie alimentaire et prévisionnelle, Ecole vétérinaire de Lyon, France) from heifers fecal samples. This strain was selected due to it being a non-pathogenic variant of *E. coli* O157:H7 (wild type strain). The pathogenicity of the bacteria was reduced due to the loss of *stx1* and *stx2* [48].

### 2.5. Bacterial Stock and Working Cultures

Stock cultures of *E. coli* were stored at −80 °C in a freezer mix, which was composed of a sterilised salt solution containing a mixture of autoclaved 12.6 g L^−1^ di-potassium hydrogen phosphate, 3.6 g L^−1^, potassium di-hydrogen phosphate, 0.9 g L^−1^, tri-sodium citrate 1.8 g L^−1^ ammonium sulphate and 300 g L^−1^ glycerol combined with a litre sterilised solution of 1.8 g L^−1^ magnesium sulphate [49]. In preparation for the cleaning assays, cultures of *E. coli* were prepared by inoculating *E. coli* onto Tryptone soya agar (TSA), at 37 °C overnight. A single colony of *E. coli* was inoculated into 10 mL of Tryptone soya broth (TSB) and incubated at 37 °C overnight. One hundred microlitres of overnight culture was inoculated into 100 mL TSB and incubated at 37 °C for 18 h with shaking (200 rpm). Following incubation, the bacterial cells were harvested by centrifuging at 1721× *g* for 10 min, washed once, and re-suspended in sterile distilled water using a vortex mixer for 30 s. The suspension was centrifuged at 1721× *g* for 10 min and the cells were resuspended to an optical density (OD) of 1.0 (±0.1) at 540 nm in sterile distilled water. This corresponded to ca. 1.88 ± 0.22 × 10^8^ CFU mL^−1^.

### 2.6. Meat Exudates

The production of meat exudates was adapted [50]. Commercially available, fresh rolled beef brisket was cut into 10 mm × 10 mm pieces, placed in a stainless steel tray and covered in aluminium foil. The meat was covered by another stainless steel tray and weighed down with 8.4 kg of stainless steel sheets and frozen at −20 °C for 24 h. The diced meat pieces were defrosted at room temperature, and the meat exudate produced was collected and stored at −20 °C until use.

### 2.7. Cleaning Assays

The substrata were inoculated with a bacterial/meat exudate mixture and dried in a microbiological class 2 cabinet. For the bacterial/meat exudate mixture, 100 µL of bacteria and 100 µL of meat exudate was placed into an Eppendorf tube, vortexed for 5 s and 10 µL of the preparation was pipetted onto the substratum, spread across the surface with a sterile plastic spreader, and dried in a class 2 flow hood at room temperature. A crockmeter was used for the wipe clean method to ensure that each wipe across the stainless steel surface was standardised. The substrata were placed on the steel specimen stage and a 45 mm × 45 mm piece of blue wipe cloth was folded and attached to the 16 mm diameter test finger. Sterile distilled water (1 mL) was pipetted onto the cloth and the hand crank was turned to simulate one wipe. The wipe cycles compromised one, five, or ten repeats. Following each cleaning cycle, the substrata were dried for 2 h in a class 2 microbiological cabinet. Three replicates were taken at each cleaning cycle point for each surface, and for each direction of clean (along or perpendicular to the linear features).

Following the cleaning assays, the percentage coverage of the bacteria and meat extract retained on the surfaces per field of view was analysed following differential staining and epifluorescence microscopy.

### 2.8. Preparation of Stains

[9-(2-carboxyphenyl)-6-diethylamino-3-xanthenylidene]-diethylammonium chloride (Rhodamine B) was prepared as a stock solution of 0.1 g mL^−1^ in ethanol (absolute) and used at a working concentration of 0.1 mg mL^−1^. 4′, 6-diamidino-2-phenylindole (DAPI) was prepared as a stock solution of 0.3 g mL^−1^ in sterile distilled water and used at a working concentration of 0.1 mg mL^−1^. Prior to use, the stains were refrigerated (4 °C) and stored in a dark environment.

### 2.9. Differential Staining of Meat Exudate and E. coli

A dual staining procedure was conducted as described previously [51]. Ten microlitres of DAPI was added to the samples and spread across the surface using a sterile plastic spreader to detect the bacteria and then 10 µL of Rhodamine B was applied to the substrate in the same manner to detect the retained meat extract [51]. Following staining, the samples were dried in the dark at room temperature in a microbiological class 2 flow hood.

The samples were viewed, and images obtained using an epifluorescence microscope with black and white digital camera and a Cell F Image Analysis package to measure the percentage coverage of the area of the stained material and to determine the percentage surface coverage of the bacteria and organic material. A filter wavelength of 330–380 nm was used to detect the DAPI stained cells, and a 590–650 nm filter was used to detect the Rhodamine B stained organic material. The retained material on the surfaces was measured using percentage coverage of the field size for randomly selected areas across the test substratum. Each of the three samples had 15 areas independently selected and analysed for the percentage coverage of bacteria and meat extract (*n* = 45).

### 2.10. Statistical Analysis

Statistical analysis was conducted by performing two-way ANOVA coupled with Tukey’s multiple comparison tests for post hoc analysis using GraphPad Prism (version 8.4.2; GraphPad Software, San Diego, CA, USA) to determine significant differences at a confidence level of 95% (*p* < 0.05). Error bars represent the standard error of the mean. Asterisks denote significance, * *p* ≤ 0.05, ** *p* ≤ 0.01, *** *p* ≤ 0.001, and **** *p* ≤ 0.0001.

## 3. Results

**Figure 1:**
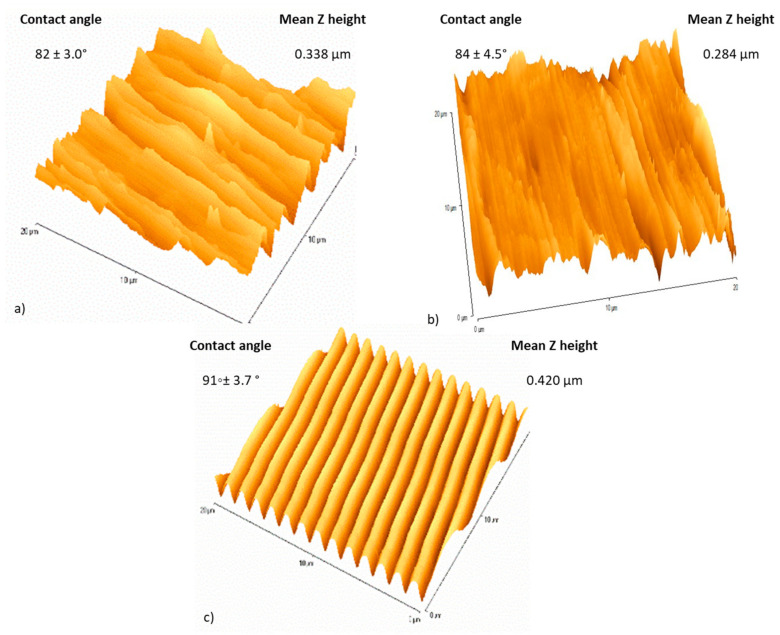
Atomic force microscopy (AFM) demonstrating the (**a**) fine polished stainless steel (FPSS), (**b**) titanium-coated fine polished stainless steel (TiFP), and (**c**) titanium coated regular linear featured surface (RG) (*n* = 15).

Three surfaces were prepared to determine the effect of a linear surface topography (irregular and regular), and defined surface chemistry (stainless steel and titanium) on the removal of bacterial and meat exudate using a wipe clean assay. Atomic force microscopy (AFM) of the fine polished stainless steel (FPSS), titanium coated fine polished stainless steel (TiFP), and the regular linear featured titanium coated surface (RG) revealed that the surface features of the FPSS and TIFP surfaces demonstrated irregular, linear topographies. The Z height of the TiFP surface (Figure 1b) was higher than the FPSS surface (0.338 ± 0.017 µm and 0.284 ± 0.014 µm, respectively) (Figure 1a). Regular linear features were evident on the titanium coated surface (RG) and the z height of the titanium coated regular surface was 0.420 ± 0.021 µm. The FPSS demonstrated valley widths of ~1 µm to 5 µm, whilst the TiFP demonstrated valley demonstrated valley widths of ~0.5 µm to 5 µm. The RG surface demonstrated valley widths of 1.02 µm. The contact angles of the three surfaces were 82 ± 3°, 84 ± 4.5°, and 91 ± 3.7° for the FPSS, TiFP and RG surfaces, respectively, and this indicated that the FPSS and TiFP were marginally more wettable than the RG surface.

**Figure 2:**
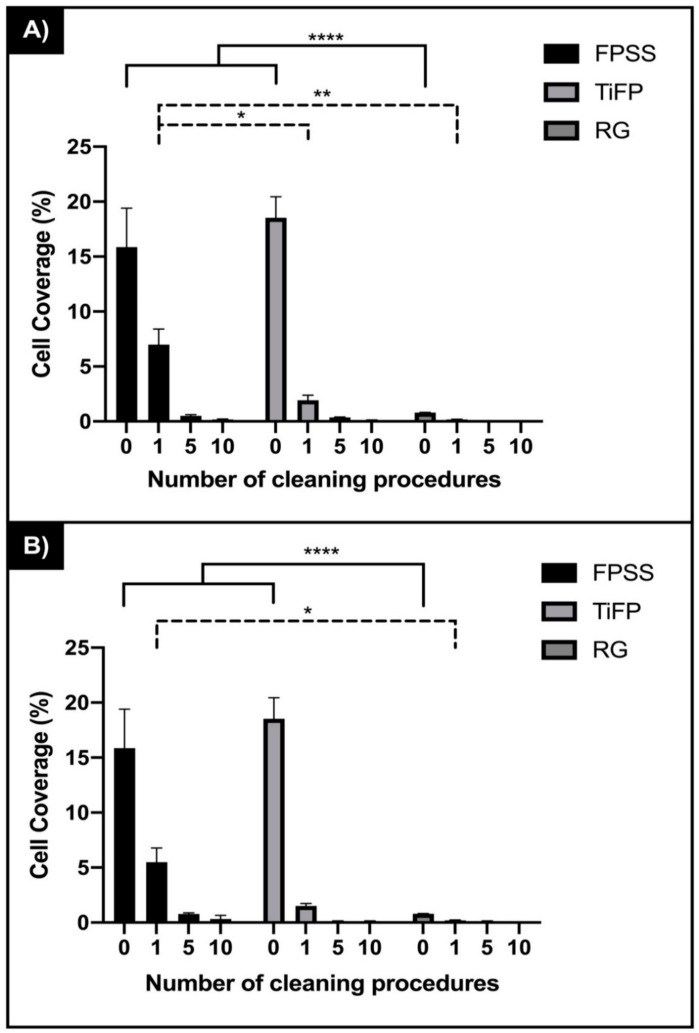
Percentage coverage bacteria retained on fine polished stainless steel (FPSS), titanium polished stainless steel (TiFP) and the regular linear featured titanium coated surface (RG) surface following 0, 1, 5 and 10 cleans (**A**) along the direction and (**B**) perpendicular to the surface features (*n* = 45). Asterisks denote significance, * *p* ≤ 0.05, ** *p ≤* 0.01 and **** *p* ≤ 0.0001.

The percentage coverage of the bacteria on the surfaces following initial fouling of the substrata before cleaning demonstrated that cells were retained in significantly higher amounts of bacteria on the FPSS (15.86%) or TiFP (18.52%) compared to the linear finished RG surface (0.81%) (*p* < 0.0001) (Figure 2).

Following one clean, fouling of the surfaces with different features and chemistries (FPSS, TiFP, RG), the amount of bacteria when cleaned along the linear features was significantly reduced (FPSS 6.98%; TiFP 1.91%; RG 0.17%) (*p* < 0.0001) (Figure 2a), whereas following one clean in the direction perpendicular to the linear features, there was only a significant difference in the amount of cells removed from the FPSS and RG surfaces (FPSS 5.49%; TiFP 1.51%; RG 0.21%) (*p* > 0.05) (Figure 2b). After five or 10 cleans, there was no significant difference in the amount of bacteria retained when the surfaces was cleaned along or perpendicular to the surface features (FPSS 6.98%, 5.49%; TiFP 1.91%, 1.51%; RG 0.17%, 0.21%) (*p* > 0.05). Overall removal of the cells from the surfaces in the direction of the linear features or perpendicular to the linear features demonstrated the same trend whereby the FPSS surface retained more bacteria than the TiFP surface, and the lowest amounts of bacteria was retained on the RG surface (Figure 2a,b).

**Figure 3:**
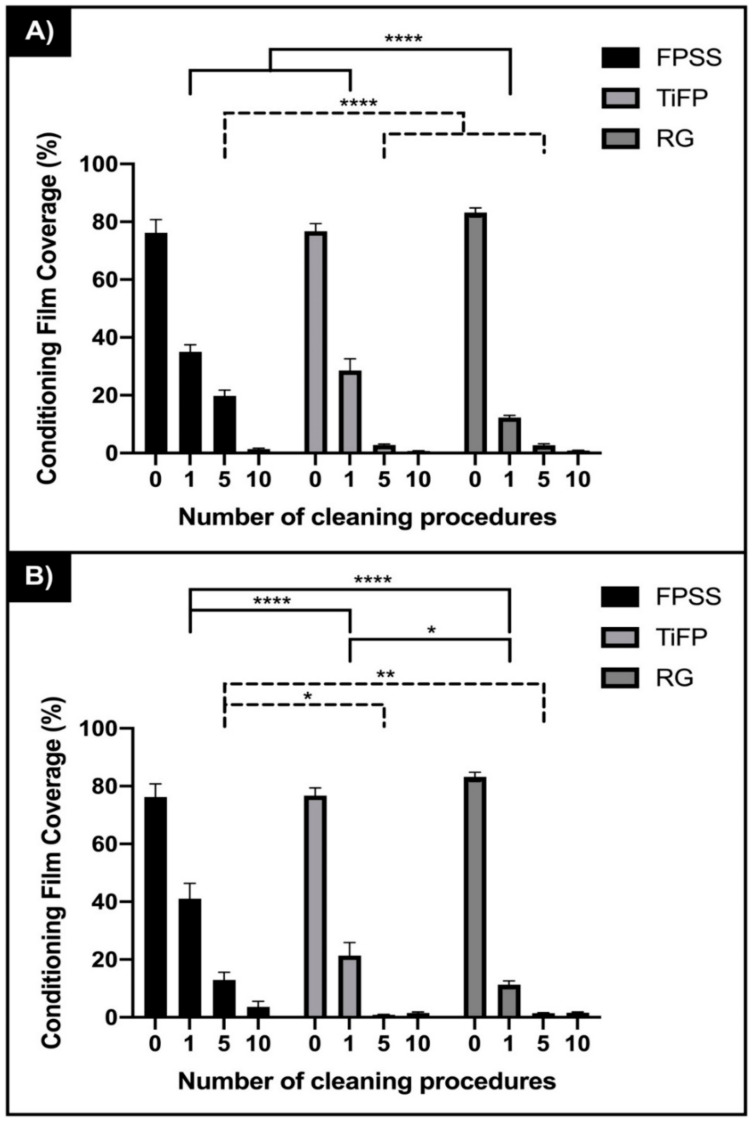
Percentage coverage of meat exudate retained on fine polished stainless steel (FPSS), titanium polished stainless steel (TiFP) and the regular linear featured titanium coated surface (RG) surface following 0, 1, 5 and 10 cleans (**A**) along the direction and (**B**) perpendicular to the surface features (*n* = 45). Asterisks denote significance, * *p* ≤ 0.05, ** *p* ≤ 0.01 and **** *p* ≤ 0.0001.

Detection of the meat exudate on the surfaces following the initial application demonstrated no significant differences in the amount of conditioning film retained on the different surfaces (FPSS, 76.2%; TiFP, 76.67% and RG, 83.20%) (*p* > 0.05) (Figure 3). The meat exudate was increasingly removed from the surfaces with increased number of cleans and this was evident for all surface types (Figure 3a,b). Following one and five cleans, there was a significant difference in the amount of meat exudate removed from the surfaces when cleaned along the linear features (*p* < 0.0001) and perpendicular to the linear features (*p* > 0.05). There was no significant difference in the amount of meat exudate retained on the different surfaces after ten cleans along (FPSS 1.4%, TiFP 0.7%, RG 0.9%), or perpendicular to (FPSS 3.6%, TiFP 1.5%, RG, 1.6%) the linear features (*p* > 0.05). However, when cleaned along the linear surface features, the overall trend was that most of the meat exudate was retained on the FPSS > TiFP > RG surface demonstrating the same trend as the removal of cells. When cleaned in the direction perpendicular to the linear features, the amount of meat exudate retained on the surfaces did not follow the same trend (one clean, FPSS > TiFP > RG; five cleans, TiFP > FPSS > RG; ten cleans, FPSS > RG > TiFP).

**Figure 4:**
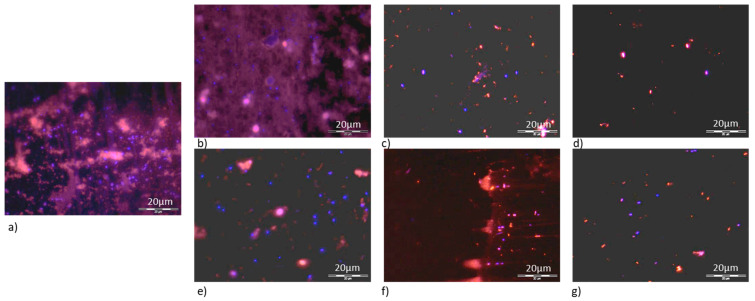
Meat exudate (red) and *E. coli* cells (blue) remaining on titanium coated fine polished stainless steel surface (TiFP) following (**a**) pre-cleaning procedure, (**b**) one wipe clean along, (**c**) five wipe cleans along, (**d**) ten wipe cleans along and (**e**) one wipe clean across, (**f**) five wipe cleans across and (**g**) ten wipe cleans across. Scale bar: 20 µm. Differential staining was conducted to visualise bacterial cells and the meat exudate on the surfaces and an example of the images on the TiFP surfaces is demonstrated (Figure 5). Prior to the cleaning procedure, the meat exudate (red) and bacterial cells (blue) can be observed in abundance (Figure 5a). The concentration of organic material and *E. coli* declined as the number of wipe cleans increased, both in the direction of, and perpendicular to the linear surface features (Figure 5).

The amount of bacteria and meat exudate removed from the surfaces following cleaning along linear features compared to cleaning in a perpendicular direction to the linear features, demonstrated that there was no significant difference (*p* > 0.05) in the removal of cells (with the exception five cleans on the FPTi). However, the meat exudate demonstrated a different trend whereby by ten cleans, the meat exudate was significantly more removed when the surfaces were cleaned in the direction along the surface features (*p* < 0.05). This result may have occurred due to the size of the bacterial cells and organic components of the meat exudate with respect to the size of the surface features (Figure 4).

**Figure 6:**
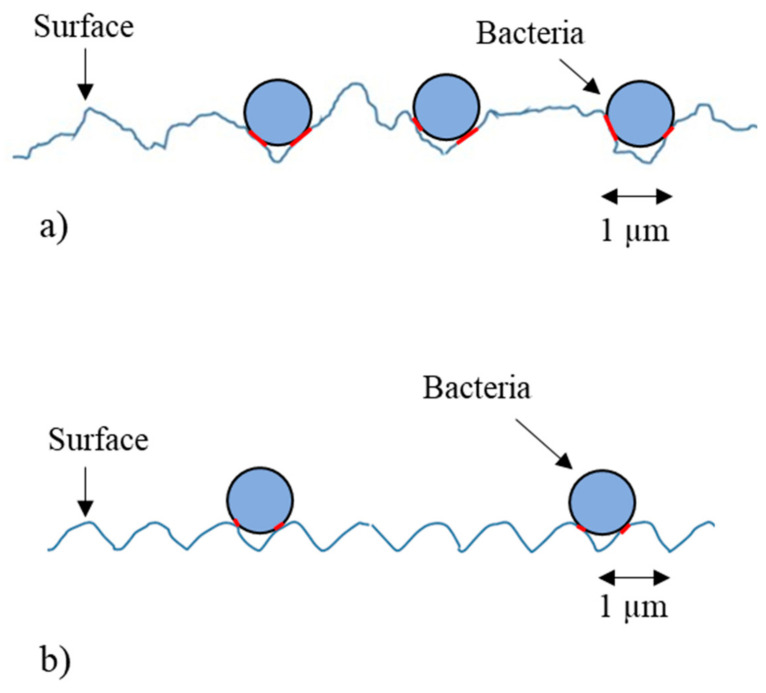
The bacteria retained on the surfaces were bound in the highest amounts on the surfaces with (**a**) irregular topographies rather than on (**b**) topographically regular surfaces.

A schematic representation of the bacteria retained, and the effect of the surface topography was produced. The bacteria were initially retained in higher amounts on the irregularly polished surfaces (FPSS and TiSS) being entrapped in the irregular surface features (Figure 6a). However, on the surfaces with regular surface features, the bacteria sat on the top, rather than inside the surface features (Figure 6b). This resulted in a lower binding of the bacteria on the surfaces and less bacterial retention.

## 4. Discussion

Product contact surfaces may contaminate meat products directly with microbial or organic material contaminants [23]. The properties of a surface play a pivotal role in bacterial and organic material retention, but nevertheless, the way in which the substrata can mediate such binding remains unclear [28,52,53].

The surfaces with regular surface topographies demonstrated clearly defined features or regular size, shape, depth, and periodicity. However, surfaces with irregular topographies, such at the fine polished stainless steel (FPSS) and the titanium coated fine polished stainless steel (TiFP), contained features of different sizes with irregular frequencies, which dependent on their size may contribute to increased or decreased bacterial binding. In this study, more cells were retained initially on the topographically irregular surfaces, which suggests that these irregular features enhanced the bacterial cell: surface interaction. The amount of bacteria retained on the irregular surface features was higher following the initial inoculation and cleans. In agreement with these findings, micropatterned topography films were utilised to determine the attachment and survival of *Escherichia coli* and *Listeria innocua* and it was demonstrated that initial bacteria attachment to the micro-pattern topography films were significantly lower in the short term [41]. In addition, after incubation with a methicillin-resistant *Staphylococcus pseudintermedius,* it was determined that bacterial biofilms tended to form in crevices [54]. However, such findings were carried out using retention and biofilm assays and were not subject to cleaning or physical forces.

Throughout this study, bacterial retention and meat exudate (e.g., the conditioning film) was quantified via differential staining and epifluorescence microscopy. The samples were prepared by adding DAPI and Rhodamine B directly to the surface and spread across the surface and dried. Although it may be considered that the methodology used in the staining method may affect the distribution of the retained material, previous studies in our laboratories have demonstrated that this is not the case since the material retained is dried onto the surface and is extremely well retained [51]. In addition, all the samples in this study were prepared using the same method; any effect which may be due to the staining process is negligible. In order for epifluorescence microscopy to be utilised effectively, samples must be prepared in a consistent manner, as was the case in this study [55].

Surfaces with features of microbial dimensions similar to those of microbial cells have been shown to promote bacterial binding, whilst the morphology of the bacterial cell can also influence such mechanisms [37,38]. All the surfaces used in this study contained surface topographies with microbial dimensions. The findings in this research demonstrated that surfaces with periodically regular dimensions decreased bacterial retention regardless of the direction of clean and removed the greatest amount of meat exudate following cleaning along the linear surface features. Although features of microbial decisions may readily retain bacteria, when a physical force is applied, it may be that the shape of the topographical feature is of importance, with the periodic regularity of the surface combined with the cell size enabling the bacteria to be easily rolled across the surface. Thus, in the context of cleaning, surface with regular topographies may enhance surface hygiene following cleaning procedures.

In addition to the surface topography, the surface chemistry may affect bacterial retention. The results demonstrated that the bacteria and meat exudate were retained in lower amounts and coverage on the titanium surfaces. In agreement with our findings, Jeyachandran et al. (2007) demonstrated that a titanium oxide film retained fewer bacteria than other materials [56]. Furthermore, Ma et al. (2008) demonstrated that the heterogeneous chemistry of a surface may provide specific contact points for bacterial retention; such points may be found on stainless steel surfaces [43]. Hence, the more homogeneous surface chemistry of the titanium coating may have resulted in a reduced number of chemically different sites, resulting in lowered bacterial and meat exudate retention. Surface wettability can interact with other surface parameters, resulting in preferential or disadvantageous bacterial retention [57,58]. In the current study, the FPSS and the TiFP surfaces were more wettable than the RG surfaces. However, the bacteria and meat extract were deposited directly onto the surfaces and hence the physicochemical effects may have been negated.

The processing of meat products results in high level of organic material remaining on food contact surfaces which conditions the underlying substrata, and it is onto the proteinaceous conditioning film to which the bacteria become retained [9,27]. It has been demonstrated that the attachment of *Pseudomonas fragi* to beef resulted in the bacteria becoming entrapped within the collagen fibres of the raw meat [59]. It has been suggested that contamination of the meat product by bacteria could be transferred to a surface, therefore thorough cleaning of surfaces and meat residues during meat production is critical to reduce the bacterial load [60]. The results from this study demonstrated that all the surfaces retained similar levels of meat extract initially, but following cleaning, the meat exudate was more difficult to remove from the surfaces with the irregular topographies. When the surfaces were cleaned in the direction along the surface features, the meat exudate was also easier to remove from the titanium coated regular surface (RG) than the titanium coated irregular surface (TiFP) or the stainless steel (FPSS). However, a clear trend on the effect of the surface properties, on the amount of meat exudate removal was not demonstrated when the surfaces were cleaned perpendicular to the linear features. Although only small amounts of organic material were retained, the difference in the trends in the effects of the surfaces properties on meat exudate retention may be due to the composition of the meat exudate, which will consist of much smaller molecules than the bacterial cells. It may be that although the bacteria can be removed by the physical force due to their larger size, the smaller organic molecules can only be pushed out of the linear features when cleaned in the direction along the linear features, as this will offer little resistance. In contrast, when the cleaning action is perpendicular to the surface features, the organic material is pushed against the wall of the surface feature where it becomes retained. This may explain the differences observed in the results.

By ten cleans, the surfaces demonstrated similar amounts of bacteria and meat exudate retained on the surfaces. One of the reasons for this is that a key component of the meat exudate is protein [50]. Protein adsorption on surfaces is a major issue in the food industry and the adsorption of proteins onto surfaces is a complex phenomenon influenced by many factors [61,62]. Protein adsorption to a surface occurs due to a range of forces and will continue until a state of equilibrium occurs [63]. It may be that as the number of cleans increased a state of equilibrium of the protein binding that occurred on the surfaces, masking the original surface properties, albeit at levels of concentrations below the detection limits of the analyses used in this study. This would effectively make the surfaces similar in terms of their characteristics.

The fouling of surfaces with proteins derived from organic foulants such as meat exudates can change the properties of a surface. A recent study conducted by Slate et al. (2019) demonstrated that the surface properties of Ti-ZrN/Ag became more hydrophilic with greater anti-adhesive properties following the introduction of a conditioning film [15]. Furthermore, the presence of a conditioning film may alter the properties of the bacterial cells themselves. When *Staphylococcus* spp. was exposed to a 10% solution of bovine serum albumin (BSA), the bacteria were demonstrated to have a reduction in their hydrophobicity and their propensity to donate electrons [64]. A linear correlation between the negative charge on the bacterial cell surface and the initial attachment to beef lean muscle and fat tissue has also been reported [65]. Such differences in the surface and bacterial properties will influence the interactions between the cell:organic material and the interface.

Standard operating procedures, which include regular cleaning, are used in the food industry to eliminate foodborne pathogens and to reduce contamination, yet despite such measures, surface contamination in food processing facilities still occurs [65]. A fundamental understanding of bacterial attachment to meat surfaces should be the basis for the development of procedures for physical removal of microorganisms that contaminate meat surfaces [11]. The determination of the removal of bacteria in the presence of meat exudate is important since although pathogens have been demonstrated to be easily destroyed by commercial sanitizers in water, the presence of organic matter may significantly affect the function of sanitizers [27,66]. A key aspect of this work is the uneven distribution of fouling across the surface. When surfaces are tested in pristine condition, this allows for easily comparative data between laboratories. However, such methodology although comparable, does not reflect a true environmental situation. The uneven distribution of the conditioning film across the surface demonstrates that the surface in a real environment will be subjected to very different material-biological interface interactions than occur when a pristine surface is used in such studies. Hence, the use of such organic material in surface-biological interactions is imperative to understand such systems.

The results from our work demonstrated that repeated cleaning of the surfaces resulted in residual organic fouling. When meat processing plants were sampled for biofilms by placing stainless steel and cast iron chips in or on floor drains and food contact areas, it was found that biofilms were formed on the drain samples but were not formed on chips placed on food contact surfaces [67]. Gibson et al. (1999) found that bacterial attachment to surfaces in the food processing environment readily occurred; however, extensive surface colonization and biofilm formation only occurred on environmental surfaces that were not regularly cleaned [23]. In addition, surfaces that were not cleaned daily, resulted in the occurrence of biofilm formation; the bacteria established in a biofilm could not be eradicated by using one single treatment or one single detergent or disinfectant, and the most effective cleaning methods were shown to require scrubbing of the surfaces [68]. With specific regards to a wipe clean, Lopez et al. (2015) showed that using a disinfectant-wipe intervention to clean a contaminated work area that was used in the preparation of chicken fillets decreased the exposure to *Campylobacter jejuni* by 2 to 3 orders of magnitude [69]. Hence, understanding the physical actions of cleaning systems is an important factor in the maintenance of hygienic systems. The cleaning process throughout the food industry is in debate over the best methods, equipment, monitoring, frequency, benchmarks, and standards to be used [70]. Thus, it is important to understand the effects that surface properties have on the cleaning efficacy of the substrata.

## 5. Conclusions

This work demonstrated that more bacteria were retained in higher amounts, initially on the stainless steel (FPSS) and titanium coated surfaces with the irregular topographies (TiFP). With subsequent cleaning, the amount of bacteria decreased and was most easily removed from the surfaces that had regular surface features and/or were titanium coated. The direction of cleaning (along or perpendicular to the linear features of the surface) did not have an effect on the amount of bacteria but did affect the amount of meat exudate retained whereby surfaces cleaned along the linear features removed more organic material. After ten cleans, the bacteria and meat exudate retained on the surfaces was not significantly different and suggested that a steady state of the surface properties had been reached. This study highlights the importance of surface properties and cleaning method selection to be utilised within the meat production industry to reduce microbial contamination and surface biofouling.

## Figures and Tables

**Figure 5:**
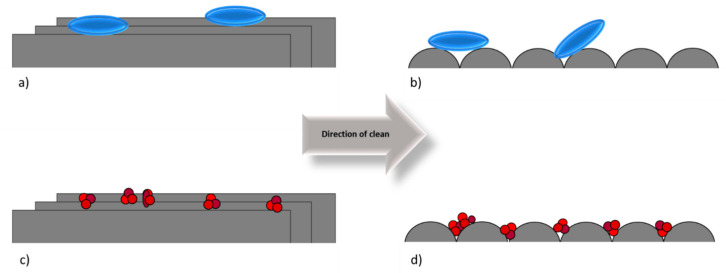
Schematic demonstrating how (**a**,**b**) the size of the bacteria (cylindrical) and (**c**,**d**) meat exudate (circles) influenced the efficacy of cleaning in the (**a**,**c**) direction of cleaning along the linear surface features or (**b**,**d**) in a direction perpendicular to the surface features.

## Data Availability

The datasets generated during the current study are available from the corresponding author on reasonable request.
